# Dispersion of Natural Airborne TiO_2_ Fibres in Excavation Activity as a Potential Environmental and Human Health Risk

**DOI:** 10.3390/ijerph18126587

**Published:** 2021-06-18

**Authors:** Sebastiano La Maestra, Francesco D’Agostini, Elisa Sanguineti, Adrián Yus González, Samanta Annis, Gaia M. Militello, Giovanni Parisi, Alberto Scuderi, Laura Gaggero

**Affiliations:** 1 Department of Health Sciences, University of Genoa, 16132 Genoa, Italy; fda@unige.it; 2 Department of Earth, Environment and Life Sciences, University of Genoa, 16132 Genoa, Italy; elisa.sanguineti@unige.it (E.S.); adrian.yus.gonzalez@edu.unige.it (A.Y.G.); samanta.annis@edu.unige.it (S.A.); gaiamaria.militello@edu.unige.it (G.M.M.); laura.gaggero@unige.it (L.G.); 3 COCIV, Via Renata Bianchi 40, I-16152 Genova, Italy; giovanni.parisi@cociv.it (G.P.); alberto.scuderi@cociv.it (A.S.)

**Keywords:** titanium dioxide, fibres, tunnel excavation, human health risk, inhalation

## Abstract

Titanium is the ninth most abundant element, approximately 0.7% of the Earth crust. It is used worldwide in large quantities for various applications. The IARC includes TiO_2_ in Group 2B as possibly carcinogenic to humans suggesting that pathological effects correlate to particle size and shape. This study case quantifies the release of natural TiO_2_ particles during mining activity, involving meta-basalt and shale lithologies in the Ligurian Alps, during excavation of the Terzo Valico as part of the Trans-European Transport Network. Type, width, length, aspect ratio, and concentration of TiO_2_ particles in needle habit were determined. The different samplings have reported that airborne concentrations in meta-basalt were 4.21 ff/L and 23.94 ff/L in shale. In both cases, the concentration never exceeds the limits established by various organizations for workers health protection. Nevertheless, TiO_2_ elongated particles, recognized as rutile, showed the dimensional characteristic of fibres, as reported by WHO. These fibres deserve particular attention because they can reach the alveolar space and trigger inflammation and chronic diseases. The results indicate that monitoring the TiO_2_ in both working environments and Ti-rich geological formations, associated with epidemiological studies, may represent a useful tool to determine the exposure risk of workers and the general population.

## 1. Introduction

Titanium is the ninth most abundant element and constitutes approximately 0.7% of the Earth crust. The main titanium minerals are rutile (TiO_2_), ilmenite (FeTiO_3_) and titanite (CaTiSiO_5_) [1].

Titanium dioxide (TiO_2_) occurs as three polymorphs, rutile and anatase, which crystallize in the tetragonal system, and brookite, which crystallizes in the orthorhombic system. Rutile is an important accessory mineral present in felsic and mafic rocks, from a low to a high metamorphic degree, as well as a detrital mineral in sedimentary rocks. Typically, rutile may incorporate different elements such as Al, V, Cr, Fe, Zr, Nb, Sn, Sb, Hf, Ta, W and U [2].

Titanium dioxide is mined worldwide in large quantities for various applications such as paints, varnishes, lacquer, paper, plastic, ceramics, rubber, and printing ink. TiO_2_ is also used in welding rod coatings, floor coverings, catalysts, coated fabrics and textiles, cosmetics, food colourants, glassware, pharmaceuticals, roofing granules, rubber tire manufacturing, and production of electronic components and dental impressions [3,4,5,6]. Titanium dioxide is chemically inert and exhibits photocatalytic activity. Therefore, the demand for TiO_2_ in the global market has increased exponentially, causing intensive extraction and consequent mineral release in the environment due to mining and industrial processing with potential environmental and human health effects. Current estimates calculate that in 2016 approximately 6.1 million tonnes of TiO_2_ were used and this will reach 7.8 million tonnes by 2022 [7]. Moreover, different anthropic activity such as tunnel excavation, material handling, placement of stones and boulders can cause dispersion of TiO_2_ into the environment in varying respirable particle-size fractions (diameter < 10 μm), including fine (diameter between 0.1 and 3 μm) and ultrafine (diameter ≤ 0.1 μm) sizes [8].

The International Agency for Research on Cancer (AIRC) includes TiO_2_ in Group 2B as possibly carcinogenic to humans suggesting that pathological effects correlate to particle size and shape [9]. Albeit the National Institute for Occupational Safety and Health (NIOSH) in 1998 acknowledges TiO_2_ as an occupational carcinogen, it does not recommend an exposure limit [10]. However, recently the NIOSH suggested airborne exposure limits of 2.4 mg/m^3^ for fine TiO_2_ and 0.3 mg/m^3^ for ultrafine (including engineered nanoscale) TiO_2_ as time-weighted average concentrations for up to 10 h/day during a 40-hour workweek [11].

Although different acts provide a quantitative risk assessment to ensure a safe and healthful working environment, currently the precise number of workers allegedly exposed to TiO_2_ is inaccurate. Moreover, the main information on human health effects was principally obtained in animals by long-term, high-dose bioassay [12]. A few epidemiological studies have evaluated the carcinogenicity of TiO_2_ exposed workers without reporting clear evidence of elevated risks of lung cancer mortality or morbidity from TiO_2_ dust expositions [13,14,15,16,17]. On the other hand, these studies report a small number of deaths from a respiratory disease other than lung cancer and no report of pneumoconiosis associated with the inhalation of TiO_2_.

A review of current toxicological data [18] has provided sufficient evidence for the carcinogenicity of titanium dioxide. Crystal dimensions may have a role in determining the level of toxicity [19]. Although the NIOSH establishes the highest acceptable concentration, different studies have demonstrated that the toxicity of TiO_2_ particles primarily depends on the physical and mineralogical characteristics of the particles, such as size, specific surface coating and crystalline form [20,21,22]. Moreover, different authors have also investigated the effect of TiO_2_ particles when inhaled or ingested [23]. In vivo studies reported that TiO_2_ particles impact genotoxicity, pulmonary inflammation, oxidative stress by reactive oxygen species (ROS) and reactive nitrogen species (RNS) generation, lung tissue damage and cell proliferation that all concur in lung cancer development [24,25].

In recent years, particular attention has been paid to monitoring TiO_2_ nanoparticle dispersion generated by industrial activities potentially harmful to human health. On the other hand, the goal of the present study was to quantify for the first time the release of natural TiO_2_ particles in needle habit during excavation processes crossing meta-basalt and shale lithologies, determining the type, width, length, aspect ratio and concentration. In addition, this study highlights the importance of EMPs’ geometric relationships overlapping with the definition of fibres by the World Health Organization (WHO).

This activity can expose workers to natural respirable Elongated Mineral Particles (EMPs) during mining, geo-engineering and infrastructure construction. Moreover, it can increase the risk of exposure in the general population caused by the environmental dispersion of such EMPs.

## 2. Materials and Methods

In order to evaluate TiO_2_ presence due to mining activity, samples from the Mt. Figogna Meta-basalts and Murta Shale formations were collected as representative lithologies crossed by the North-South oriented Terzo Valico tunnel across the Ligurian Alps, which is part of the Trans-European Transport Network (TEN-T) railway. The Mt. Figogna Meta-basalts originated in the Jurassic oceanic lithosphere and were overprinted under alpine pumpellyite-actinolite facies, developing the assemblage albite, Ca-amphibole, chlorite, epidote, pumpellyite and titanite, rutile and hematite as accessory mineral phases.

The Murta Shales is a low-grade metamorphic unit of the Ligurian Alps, made of dark grey shales with pervasive schistosity characterised by graphite-sericite alternating micro-lithons. Shales are locally rich in graphite or show decimetre-metre thick intercalations of micritic limestone and micaceous limestone. The mineral assemblage consists of mica, calcite, quartz, chlorite, while accessory minerals are magnetite or pyrite and rutile.

Five rock samples were gathered from the excavated rock front for each lithotype, and 10 airborne filter samples were obtained during excavation work. A total of ten rock samples and twenty airborne filters were analysed. The air sampling stations were located as close as possible to the source of dust during the excavation activity. Preparation and analyses were carried out at the Earth Environment and Life Sciences Department (DISTAV), University of Genoa, following methods prescribed for asbestos. In particular, the Italian legislative decree that regulates the protection of workers (L.D. 81/2008) refers to the method described in the Ministerial Decree (M.D.) 06/09/1994 (All. 2—Quantitative determination of airborne asbestos fibre concentrations in indoor environments), which is similar to the ISO Method 14996:2002 (ISO, 2002).

### 2.1. Rock and Airborne Sample Preparation

Airborne particulate was collected using a high flow sampling system depositing on polycarbonate membrane filters with a diameter of 25 mm and a pore size of 0.8 µm. Each sampling lasted for 8 h. Air was aspirated at a flow rate of 6 L/min, a total of 3000 L and was partitioned on 6 different filters due to a high concentration of environmental dust [26].

Differently representative aliquots (100 g) of rock samples were ground to a grain size of 100 μm with a Vibratory Disc Mill Retsch RS200 equipped with stainless steel grinding jars. Milling conditions were 25 s at 900 rpm and 15 s at 900 rpm for meta-basalt and shale samples, respectively. About 10 mg of the obtained powders were suspended in 100 mL of deionised water, and the obtained suspension was sonicated for 10 min. Five millilitres of the suspension was deposited on a polycarbonate membrane (47 mm diameter, 0.8 μm porosity) and filtered using a vacuum filtration system, obtaining about 0.5 mg of powders.

### 2.2. Scanning Electron Microscopy (SEM) analysis

All samples were analysed by SEM (Tescan Vega 3 XML), with a W source working at 20 kV of acceleration voltage. The elemental analysis was carried out by an Energy Dispersive X-ray spectrometer (EDS, Oxford Instruments, X-Max 20 SDD detector). A portion of each polycarbonate membrane was cut, mounted on an aluminium stub using a carbon conductive tab and coated with a thin gold layer by sputtering, using a Quorum Q150T ES.

For each sample, 54 random fields corresponding to a filter surface of 1 mm^2^ were analysed by SEM-EDS at 2000× magnification. The number of TiO_2_-EMPs was counted in each field, and the length and width were measured for each particle. Particle concentration in each sample was obtained applying the quantitative determination methods for airborne asbestos fibre using the Formula (1) reported in M.D. 06/09/1994, All. 2:

(1) $$C=N_{f}\cdot \frac{1}{a\cdot N_{c}}\cdot A_{f}\cdot \frac{1}{V}$$

*C* = particle concentration
*N_f_* = total number of fibres counted
*a* = field area at 2000× (mm^2^)
*N_c_* = total number of fields examined on the filter
*A_f_* = effective collecting area of filter (mm^2^)
*V* = the volume of sampled air (L)

The number of TiO_2_-EMPs found was expressed as ff/L with confidence limits of 95%, assuming a Poisson distribution. Analytical sensitivity of the method was expressed, such as concentration, equivalent to observation of one fibre (*N_f_* = 1), and the *V*, *A_f_*, and *N_c_* function. The volume of each fibre was approximated to a cylinder, having diameter and height equal to fibre width and length, respectively. The weight of each EMP was calculated assuming an average density of the rutile (4.2 g/cm^3^) and correlated with the volume of particles. Moreover, the concentration expressed as mg/m^3^ was calculated assuming *N_f_* as the total weight of the fibres found (mg) and the volume as the volume of sampled air expressed in m^3^.

### 2.3. Micro-Raman Spectroscopy

Micro-Raman analysis was performed by a Horiba Jobin-Yvon Explora-Plus spectrometer equipped with a charge-coupled device (CCD) detector, an Nd-YAG laser (532 nm), and Olympus BX 40 optical microscope with a 100X LWD objective and a grating of 2400 grooves/mm leading to a nominal spectral resolution of about 2 cm^−1^. The spectrometer was calibrated to the silicon Raman peak at 520.5 cm^−1^. Spectrum acquisition time was 20 s averaged between two accumulations in the spectral range between 50 and 1000 cm^−1^. Analyses were carried on TiO_2_ fibres sampled on a polycarbonate membrane filter.

## 3. Results

**Figure 1:**
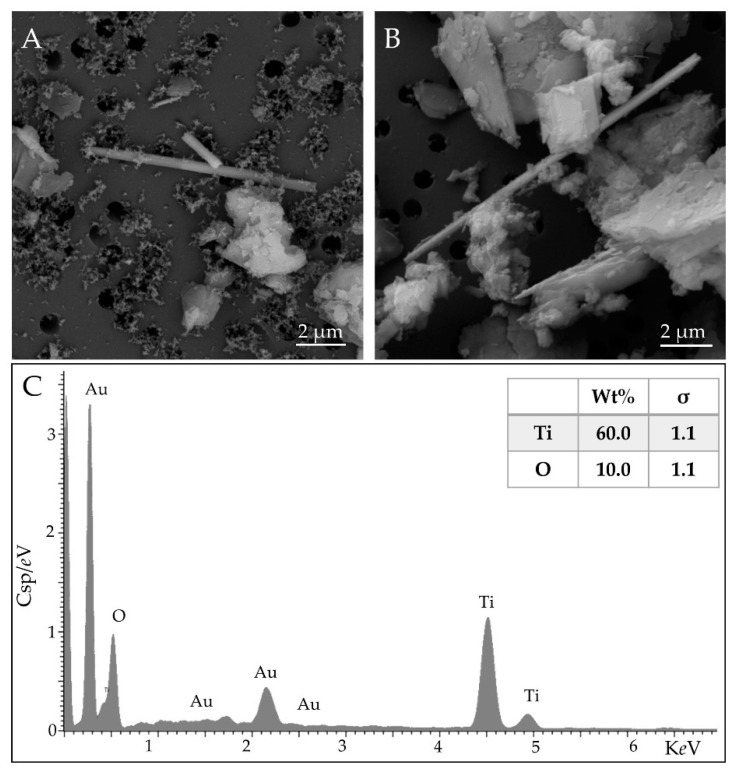
Detail of airborne rutile fibers in shale sample at 20,000× (**A**,**B**) and the representative elemental analysis (**C**). The results are shown as weight percentage (Wt%). The statistical error is displayed as sigma (σ), representing the overall confidence figure for the analysis, especially when an element is present at a low concentration. Scale bar in microphotographs; High Vacuum: 20 kV; Detector: Back Scattered Electrons and Energy Dispersive Spectroscopy.

Meta-basalts and shales represent the principal lithotypes in the monitored area. In both lithotypes, the primary source of TiO_2_ is represented by rutile, which exhibits an acicular and fibrous habit, as shown in Figure 1A,B.

**Figure 2:**
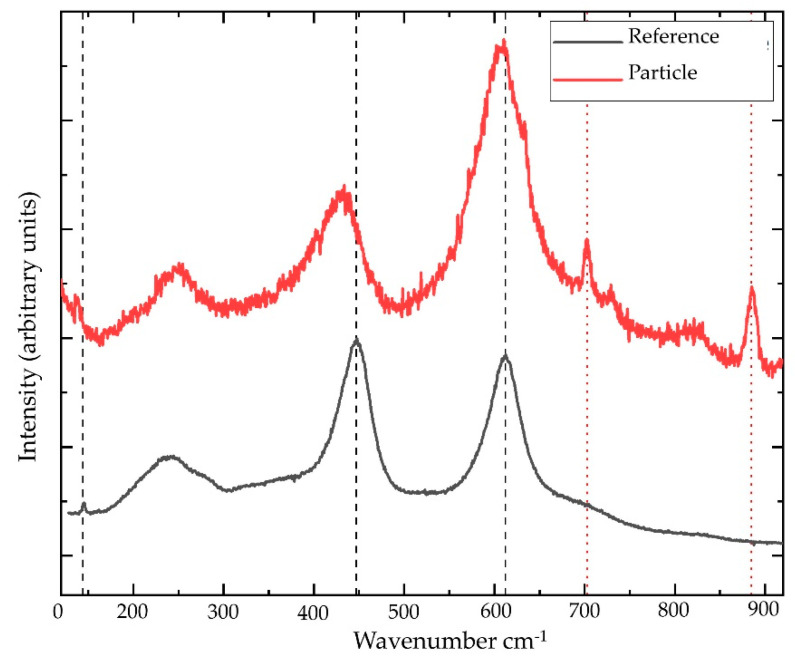
Raman spectrum of a needle (red) compared to a reference spectrum of rutile (black) from the free database Handbook of Raman spectra Lyon University (http://www.geologielyon.fr/Raman/spectrum accessed on 6 April 2021). Black dashed lines are eye-guides for the wavenumbers of the three Raman active modes in rutile (near 146, 447 and 612 cm^−1^). Red dotted lines indicate two of the main Raman peaks visible from the surrounding polycarbonate (near 702 and 884 cm^−1^).

The results obtained by µ-Raman (Figure 2) confirmed that the detected polymorph in both lithologies was rutile.

Generally, the excavation procedure generates a large amount of dust from the rocks, which, disintegrating, release tiny particles suspended in the air. The extent of dust formation, as well as persistence in the environment, are relatable to the nature of the rocks and the size of the particles together with the environmental conditions such as humidity, temperature, circulating air and gas emissions inside the tunnel.

### 3.1. TiO_2_-EMPs in Massive Rock Samples

**Figure 3:**
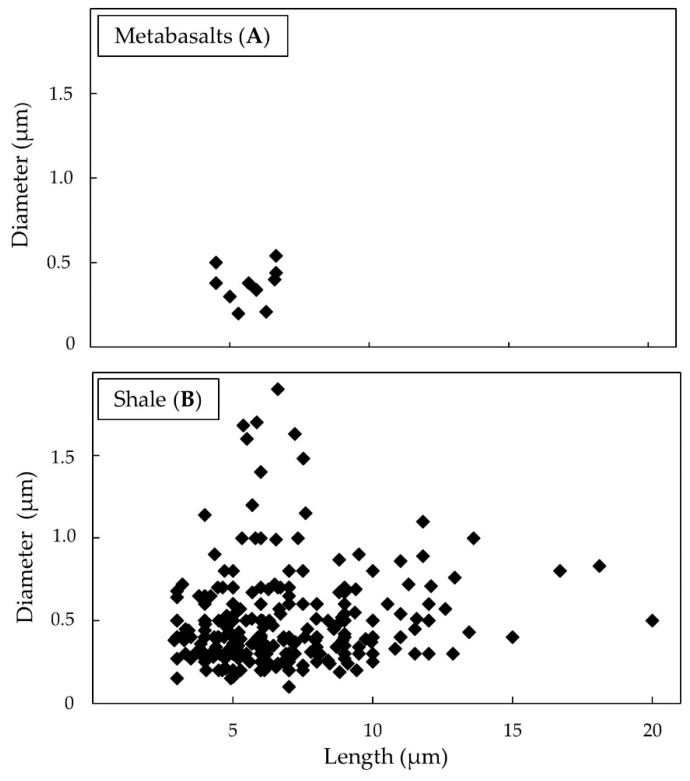
Size distribution of fibres in meta-basalt (**A**) shale (**B**) and massive samples.

The TiO_2_-EMPs found in massive meta-basalts, despite being poor, had a length from 4.5 to 6.65 µm, averaging 5.68 µm. The average diameter is 0.38 µm with values ranging from 0.21 to 0.54 µm (Figure 3A). Noteworthy, the length to diameter ratio (L/D) of fibres in both lithotypes exceed the World Health Organization (WHO) established ratio of 3:1 [27].

In massive shale samples, TiO_2_-EMPs showed a length of between 2.9 and 20 µm while the average value was 6.70 µm. All fine fibres detected had a diameter between 0.1 and 1.90 µm with an average diameter of 0.48 µm. The lengths of fibre distributions showed the following percentage: 36.84% for fibres from 2.5 to 5 µm, 33.33% between 5 to 7.5 µm, and 20.35% between 7.5 to 10 µm, respectively. The remaining 9.47% was distributed between lengths from 10 to 20 µm (Figure 3B).

**Figure 4:**
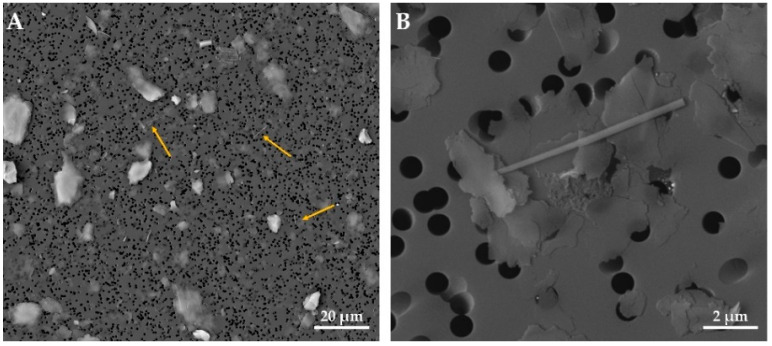
Acicular rutile from massive shale sample. Overview of the reading field at 2000× (**A**) and detail of rutile fiber at 20,000× (**B**). Scale bar in microphotographs; High Vacuum: 20 kV; Detector: Back Scattered Electrons.

Figure 4A,B shows examples of SEM analyses of rutile needle in the massive shale sample.

### 3.2. TiO_2_-EMPs in Airborne Sample

#### 3.2.1. Meta-basalts

**Figure 5:**
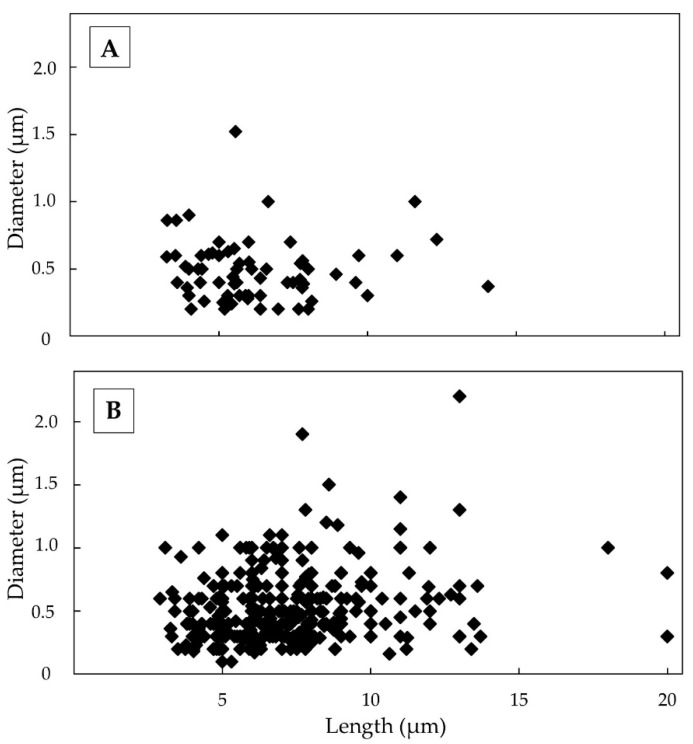
Size distribution of fibres in meta-basalt (**A**) and shale (**B**) airborne samples.

SEM observation of the airborne samples obtained during the meta-basalt excavation front exhibited a fibre distribution whose length ranges between 3.24 and 14.06 µm, with an average length of 6.23 µm. About 69 TiO_2_-EMPs were found, and of these 43.5% had a length of between 5 to 7.5 µm, while 36.2% showed a diameter lower than 0.4 µm. Moreover, 31.9% of the fibres reported a length from 2.5 to 5 µm and 18.8% from 7.5 to 10 µm, respectively. The remaining 5.8% varied between lengths from 10 to 20 µm. All fibres detected had a diameter between 0.2 and 1.6 µm with an average diameter equal to 0.48 µm and, therefore, were classifiable as fine fibres (Figure 5A). The results also indicated the presence of a variable aspect ratio where the prevalent classes were 10:1 (23.18%), 20:1 (46.38%) and 30:1 (13.04%). The meta-basalt fibres did not exceed ratios up to 40:1.

**Figure 6:**
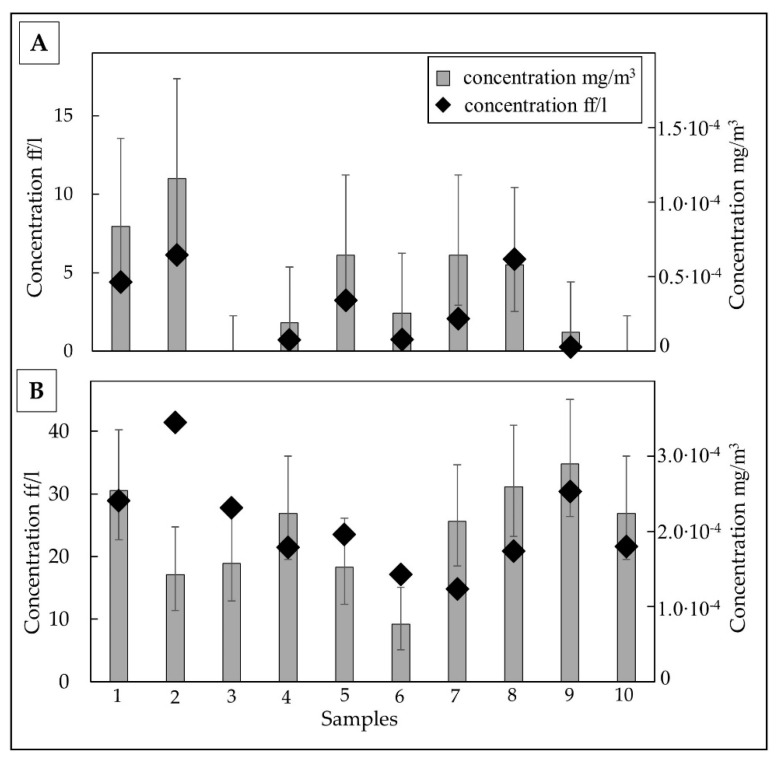
TiO_2_ elongated particle concentrations reported as mg/m^3^ (black diamond) or ff/L (grey bar) ± fiducial limits (F 95%) in airborne samples of meta-basalt (**A**) and shale (**B**).

Airborne TiO_2_ fibres concentrations in meta-basalt samples were 4.21 ff/L, ranging between 1.22 and 10.99 ff/L. The average concentration expressed as mg/m^3^ was equal to 2.47∙10^−5^ mg/m^3^ with values between 6.44∙10^−5^ and 2.85∙10^−6^ mg/m^3^. TiO_2_ fibre concentrations were expressed as both ff/L in the grey bar and mg/m^3^ in a black diamond, as reported in Figure 6A.

#### 3.2.2. Shales

SEM-EDS observations performed at 2000× magnification showed a significant number of TiO_2_ fibres (392 fibres) from airborne shale samples with a length of between 2.9 and 20 µm with an average length of 7.04 µm. The 24% of fibres reported a length from 2.5 to 5 µm, the 42.3% between 5 to 7.5 µm and the 24.7% from 7.5 to 10 µm, respectively. The remaining 8% were distributed between lengths from 10 to 20 µm. All fibres detected had a diameter between 0.1 and 2.2 µm and, therefore, were classifiable as fine fibres, with an average diameter of 0.52 µm (Figure 5B).

**Figure 7:**
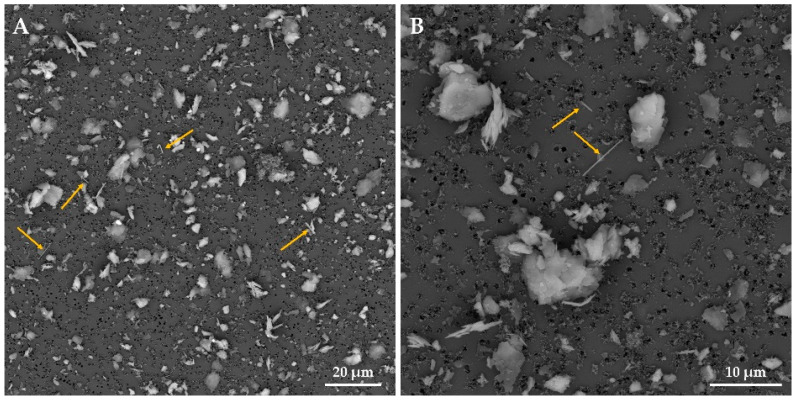
Overview of the reading field of airborne sampling from shales at 2000× (**A**) and 5000× (**B**). Scale bar in microphotographs; High Vacuum: 20 kV; Detector: Back Scattered Electrons.

A further investigation aimed at detecting the possible presence of ultrafine particles was performed under 5000× magnification (Figure 7), evidencing an additional class (15.81%) of particles with a length of between 1.6 and 2.5 µm. These fibres showed a diameter ranging between 0.1–0.5 µm. Aspect ratio shows prevalent classes 10:1 (24.23%), 20:1 (49.74%) and 30:1 (17.09%). In summary, 75.77% had an aspect ratio > 20:1 and 7.39% had an aspect ratio > 40:1. Noteworthy, a small percentage of fibres reached ratios of up to 70:1.

The average airborne TiO_2_ fibre concentration in shale samples was 23.94 ff/L, ranging between 9.16 and 34.81 ff/L. The average concentration expressed as mg/m^3^ was 2.07∙10^−4^ mg/m^3^ with values between 3.45∙10^−4^ and 1.24∙10^−4^ mg/m^3^. TiO_2_ fibre concentration was expressed both as ff/L and mg/m^3^ (Figure 6B).

## 4. Discussion

Excavation activities at Fegino and Polcevera sites (Genoa, Italia) were addressed with reference to the “Mt. Figogna Meta-basalts” and “Murta Shales” formations. The air monitoring activity performed to detect asbestos began during the different excavation phases, evidencing a significantly associated occurrence of TiO_2_-EMPs. SEM and µ-Raman analysis performed on membrane filters indicated that, in both meta-basalts and shales, the TiO_2_ polymorph was rutile. TiO_2_ crystals occurred with fibrous habit principally as single needles, in some cases as elbow-shaped twinned fibres. In shales, TiO_2_ fibres are often included in mica or chlorite. All fibres have homogeneous size distribution despite higher concentrations in shales. Therefore, the features of TiO_2_ fibres such as shape and size and the textural properties of the isolated or enclosed examples, together with the mechanical stress induced by the excavation process, may not influence their dimensions, concentrations, and dispersion in the environment. The different concentration values are likely linked to the primary (detrital) or secondary (metamorphic) rutile origin and its high mechanical resistance.

The majority of all measured rutile crystal sizes fell in the dimension interval established by WHO for fibre classifications following the geometric requisites criteria of fibres (length greater than 5 µm, diameter smaller than 3 µm, and length diameter ratio equal to or greater than 3:1) [27]. In particular, the percentage of EMPs that covered the geometrical aspect of fibre were 80% and 64.32% for meta-basalts and shale in massive rock samples, respectively. In the airborne samples, the percentage was 71% for meta-basalts and 85% for shale. Detailed counting at 5000× has reported the occurrence of elongated rutile particles with length <5 µm, assessing the presence of ultrafine TiO_2_ particles.

These fibres, if dispersed in the environment, can be inhaled or ingested, playing a crucial role in the etiopathogenesis of different diseases. Although the toxicity of TiO_2_ particles was low, lung tumours were demonstrated in animal study after two years of exposure to a high concentration of fine particles [12]. However, TiO_2_ fibres can cross the gastrointestinal tract of rats, translocating in peripheral tissue and lymph node, respectively [28]. Rutile is therefore considered a chemically inert mineral that can have a carcinogenic effect attributed to lung overload.

The American Conference of Governmental Industrial Hygienists has assigned TiO_2_ fine particles a threshold limit value of 10 mg/m^3^ as a time-weighted average for a typical 8 h workday and a 40-h workweek [29]. Further indications from Occupational Safety & Health Administration (OSHA) indicate the permissible exposure limit for TiO_2_ fine particle concentrations at 15 mg/m^3^ [30]. Our case study found average concentrations of TiO_2_ in airborne samples equal to 2.47 ± 10^−5^ mg/m^3^ for meta-basalt and 2.07 ± 10^−4^ mg/m^3^ for shale, values significantly lower than those reported above.

This monitoring carried out in the indoor working environment reported values below the limit indicated by the control agencies referred to above. However, it is important to underline the high abundance of TiO_2_ fibres observed in the analysed samples. Although several studies have been carried out to determine the respiratory carcinogenicity of TiO_2_, the spherical shape of the mineral has been principally studied. This does not allow correlation of the significance of the impact of shape to disease development. The shapes of TiO_2_ could be divided into two main types, spherical and fibrous particles. It is plausible that TiO_2_-EMPs could have similar behaviour to asbestos fibres [31]. The carcinogenic mechanism of the fibres is traced back to their capacity to translocate to the pleura inducing malignant mesothelioma. Generally, fibrous particles induced lung tumours through persistent inflammation due to incomplete phagocytosis and a release of ROS and RNS, such as different proinflammatory cytokines [32,33]. Bio-persistence and poor solubility are believed to be the most critical factors in this toxicity.

The TiO_2_-EMPs observed in airborne samples from meta-basalts and shale generally follow the size of fibres defined by WHO. Due to their small diameter (<3 µm), these can be classified as fine respirable fibres, able to reach the alveolar environment, triggering inflammatory and recruitment of inflammatory cells. In fact, the cellular damage induced by fibrous particles may occur both through direct nucleus damage, due to the penetration of EMP breaking the structure of DNA and generation of ROS, caused by metal species incorporated in rutile, such as Fe, Cr, and V able to trigger a redox-active reaction [34].

The inclusion of TiO_2_ in group 2B is based on rat studies and chronic inhalation exposure. In this regard, different debates are ongoing such as the representativeness of rat lungs in comparison to those of humans. Moreover, it is not clear if TiO_2_–EMPs reactivity is due to the chemical or physical (i.e., particle) nature. In fact, the biological mechanism leading to carcinogenicity induced by inhalation and ingestion of particles is only partly understood [12,35]. Different studies have suggested that TiO_2_ might induce or promote colon tumours via inflammation and ROS production [36,37].

Currently, available information does not allow conclusions on the relationship between specific physico-chemical characteristics of TiO_2_ particles and the mechanisms triggering pathological events [38]. Therefore, future studies should characterize particles thoroughly and focus on specific characteristics such as crystal structure, size, surface coating, and reactivity of TiO_2_ in relevant cell types to investigate whether all forms of TiO_2_ should be considered to be of equal toxic potential.

Air monitoring activity of the asbestos carried out at the Polcevera and Fegino sites highlighted a number of rutile fibres deriving from the process of excavation. These findings indicated the possible contamination of the environment, mainly due to the presence of rutile in the host rocks and anthropic activities performed at these sites. The EMPs released in the environment represent a secondary source of contaminants that can be conveyed in aquifers by percolating rainwater.

Jani et al. [28] reported rutile particle (sized 0.5 µm) uptake from the rat gastrointestinal tract and subsequent translocation to systemic organs after oral administration. TiO_2_ particles, especially nanoparticles, have been demonstrated to cross the cellular barrier, both in vivo and in vitro, although sometimes translocation was mild and did not finalize in any significant systemic effects. Inflammation and enhanced initiation or promotion of colorectal carcinogenesis were observed but not confirmed [39].

Moreover, non-inhaled rutile fibres, despite their high specific weight, showed aerodynamic characteristics and persistence, remaining in the atmosphere for long periods, dispersing over long distances and contaminating different sites [40,41].

## 5. Conclusions

The present study results show that rutile environmental dispersion is mainly due to anthropological activity. TiO_2_ fibres in the airborne samples have already been highlighted in our monitoring activities aimed at detecting the presence of asbestos minerals from the Polcevera and Fegino sites. The different samplings have reported that concentrations of TiO_2_ never exceed the limits established by different organizations for workers’ health protection. Though the AIRC has included the TiO_2_ in group 2B, it is noteworthy that various epidemiological studies do not support this classification. In this regard, new environmental and human biomonitoring studies would be necessary to facilitate the long-term risk assessment caused by inhalation or ingestion of elongated particles. This information may be helpful to increase scientific knowledge and promote reduced TiO_2_ human exposure. Biomonitoring studies addressing the risk caused by TiO_2_ inhalation in different work activities such as mining are currently lacking.

Contrarily to asbestos which has a proven harmful effect on human health, specific legislation on TiO_2_ monitoring is lacking. However, the data discussed in this paper demonstrated that most TiO_2_ elongated particles, recognized as rutile, had the dimensional characteristic of fibres, as reported by WHO. In particular, a fraction of the fibres fall is fine, able to reach alveolar space and trigger an inflammation process that can lead to pneumoconiosis. Pneumoconiosis may result in several complications such as tuberculous superinfection, chronic bronchitis, emphysema, respiratory failure, chronic pulmonary hypertension, and tumours of the respiratory system, especially in smokers, enhanced by a significant synergic effect.

The importance of monitoring TiO_2_ in both working environments and naturally hazardous areas, such as stream sediments and internal waters across Ti-rich geological formations, associated with epidemiological studies, may represent a useful tool to determine the exposure risk of workers and the whole population.

## Data Availability

Not applicable.
